# Potential Value of Expiratory CT in Quantitative Assessment of Pulmonary Vessels in COPD

**DOI:** 10.3389/fmed.2021.761804

**Published:** 2021-10-14

**Authors:** Xianxian Cao, Xiaoyan Gao, Nan Yu, Meijuan Shi, Xia Wei, Xiaoqi Huang, Shudi Xu, Jiantao Pu, Chenwang Jin, Youmin Guo

**Affiliations:** ^1^Department of Radiology, The First Affiliated Hospital of Xi'an Jiaotong University, Xi'an, China; ^2^Department of Diagnostic Imaging, National Cancer Center/National Clinical Research Center for Cancer/Cancer Hospital, Chinese Academy of Medical Sciences and Peking Union Medical College, Beijing, China; ^3^Medical Imaging Center, Shaanxi Provincial People's Hospital, Xi'an, China; ^4^Department of Radiology, The Affiliated Hospital of Shaanxi University of Traditional Chinese Medicine, Xianyang, China; ^5^Department of Radiology, The Second Affiliated Hospital of Xi'an Jiaotong University, Xi'an, China; ^6^Department of Respiratory Medicine, The Ninth Hospital of Xi'an Affiliated Hospital of Xi'an Jiaotong University, Xi'an, China; ^7^Department of Radiology, The Affiliated Hospital of Yan'an University, Yan'an, China; ^8^Departments of Radiology and Bioengineering, University of Pittsburgh, Pittsburgh, PA, United States

**Keywords:** chronic obstructive pulmonary disease (COPD), computed tomography, intrapulmonary vessels, inspiratory, expiratory

## Abstract

**Objective:** To investigate the associations between intrapulmonary vascular volume (IPVV) depicted on inspiratory and expiratory CT scans and disease severity in COPD patients, and to determine which CT parameters can be used to predict IPVV.

**Methods:** We retrospectively collected 89 CT examinations acquired on COPD patients from an available database. All subjects underwent both inspiratory and expiratory CT scans. We quantified the IPVV, airway wall thickness (WT), the percentage of the airway wall area (WA%), and the extent of emphysema (LAA%_−950_) using an available pulmonary image analysis tool. The underlying relationship between IPVV and COPD severity, which was defined as mild COPD (GOLD stage I and II) and severe COPD (GOLD stage III and IV), was analyzed using the Student's *t*-test (or Mann-Whitney *U*-test). The correlations of IPVV with pulmonary function tests (PFTs), LAA%_−950_, and airway parameters for the third to sixth generation bronchus were analyzed using the Pearson or Spearman's rank correlation coefficients and multiple stepwise regression.

**Results:** In the subgroup with only inspiratory examinations, the correlation coefficients between IPVV and PFT measures were −0.215 ~ −0.292 (*p* < 0.05), the correlation coefficients between IPVV and WT_3−6_ were 0.233 ~ 0.557 (*p* < 0.05), and the correlation coefficient between IPVV and LAA%_−950_ were 0.238 ~ 0.409 (*p* < 0.05). In the subgroup with only expiratory scan, the correlation coefficients between IPVV and PFT measures were −0.238 ~ −0.360 (*p* < 0.05), the correlation coefficients between IPVV and WT_3−6_ were 0.260 ~ 0.566 (*p* < 0.05), and the correlation coefficient between IPVV and LAA%_−950_ were 0.241 ~ 0.362 (*p* < 0.05). The multiple stepwise regression analyses demonstrated that WT were independently associated with IPVV (*P* < 0.05).

**Conclusion:** The expiratory CT scans can provide a more accurate assessment of COPD than the inspiratory CT scans, and the airway wall thickness maybe an independent predictor of pulmonary vascular alteration in patients with COPD.

## Introduction

Chronic obstructive pulmonary disease (COPD) is very prevalent worldwide and carries high mortality and morbidity rates (1, 2). Among COPD patients, 30–70% have clinically significant pulmonary vascular disease (3–5). The major vascular alterations are vascular remodeling and vasoconstriction caused by emphysema and/or hypoxemia, and often cause pulmonary hypertension (6, 7). There are investigations showing that pulmonary vascular alterations were found in patients with mild COPD, even in non-smokers with normal lung function (8, 9). All these suggest that pulmonary vascular alterations may persist throughout the entire progress of COPD, and it is important to develop methods to quantitatively assess the pulmonary vascular alterations in COPD.

The high-resolution characteristic of computed tomography (CT) makes it possible to visualize very detailed lung structures and quantify a variety of lung abnormalities, such as emphysema, airway remodeling, and pulmonary vascular alterations in COPD (10, 11). There have been investigative efforts made to quantitatively assess pulmonary vascular alterations in COPD. Matsuoka et al. (12) proposed the total cross-sectional area (CSA) of small pulmonary vessels as an index of pulmonary vascular alterations. They reported that %CSA <5 mm^2^ had a significant correlation with forced expiratory volume in 1 s (FEV_1_) and FEV_1_/forced vital capacity (FVC) as well as %LAA_−950_ in severe COPD. Previous studies (13–15) have demonstrated that there were quantitative pulmonary vascular features, such as the percentage of total vessel area and the number of small vessels, closely associated with survival and PFT measures in COPD patients. It is notable that most of the available investigations about pulmonary vascular alternation were limited to the inspiratory CT scans. Although there are studies (16–18) demonstrating the unique value of expiratory CT examinations in assessing COPD, it is unclear whether the expiratory CT scans have any advantage over inspiratory CT scans in assessing pulmonary vascular alternation.

In this study, we proposed to quantify the intrapulmonary vascular volume (IPVV) depicted on CT images in COPD patients. The objective is to study whether pulmonary vascular alternations in COPD subjects are associated with emphysema extent, pulmonary functions, and airway abnormalities, and to determine which parameter can be used as predictor of IPVV in COPD patients. In particular, we performed the analyses on both inspiratory and expiratory CT scans, aiming to clarify the potential of expiratory CT examinations in assessing pulmonary vascular alternations in COPD. For this purpose, we established a dataset consisting of 89 paired inspiration-expiration CT scans. A detailed description of our dataset, methods, and experimental results follows.

## Materials and Methods

### Study Population

We retrospectively identified 92 patients from the “Digital Lung” Respiratory Disease Evaluation System and Diagnostic Criteria (201402013). These subjects were diagnosed with COPD and underwent both inspiratory and expiratory CT examinations. COPD was diagnosed on the basis of past history, physical examination, and spirometry data by following the Global Initiative for Chronic Obstructive Lung Disease (GOLD) (1) diagnostic criteria (FEV_1_/FVC <70% bronchodilators inhaled). Among the collected subjects, three were excluded, because of the involved issues: (1) concomitant lung diseases such as interstitial lung disease, lung cancer, infectious pneumonia, and pulmonary tuberculosis; (2) previous lung surgery; (3) insufficient CT quality of analysis; and (4) unable to complete the pulmonary function test. As a result, we have 89 subjects involved in this study and the demographics information was summarized in Table 1. All subjects were divided into subjects with mild COPD (GOLD I and II, *n* = 43) and subjects with severe COPD (GOLD III and IV, n=46) for comparison of IPVV. This retrospective study was approved by the Chinese Clinical Research Registry (Grant No.: ChiCTR-OCH-14004904), and written informed consent was obtained from all subjects.

**Table 1:** Patient Characteristics and PFT results in the COPD subjects.

**Characteristic**	**COPD subjects (*n* = 89)**
Age (years)	63.6 ± 9.4
Sex, %female	19 (21.35%)
BMI (kg/m^2^)	22.76 ± 3.59
GOLD stage I:II:III:IV	12:31:28:18
FEV_1_/FVC%	51.45 ± 9.75
FEV_1_%	47.00 (32.85)

*BMI, body mass index; FEV_1_/FVC, ratio of forced expiratory volume in 1 s to forced vital capacity; FEV_1_, percentage predicted forced expiratory volume in 1 s*.

### Pulmonary Function Tests

All subjects underwent spirometry according to American Thoracic Society/European Thoracic Society guidelines (19). PFT measurements included forced expiratory volume during the first second of exhalation (FEV_1_) percent to the predicted value (FEV_1_%predicted) post inhalation of 200 μg salbutamol, FEV_1_/forced vital capacity ratio (FEV_1_/FVC), the ratio of residual volume to total lung capacity(RV%TLC) and the diffusing capacity for carbon monoxide (DL_CO_). Referring to previous studies (20, 21), we only used the FEV_1_% predicted and FEV_1_/FVC in the subsequent analysis in this study.

### CT Scan Acquisition

The CT examinations were performed at full inspiration and expiration states for the involved subjects in the supine position using 64-slice multi-detector CT scanners (SOMATOM Definition AS; Siemens, Erlangen, Germany). All subjects were given breathing training prior to examination. The scan parameters were as follows: tube voltage: 100 or 120 KV tube current, autoexposure control, exposure time: 0.5 s, and the matrix size: 512 × 512 pixels. Images were reconstructed with a 1 mm slice thickness (with 0.625 mm overlap) using a standard kernel algorithm.

### Image Processing

**Figure 1:**
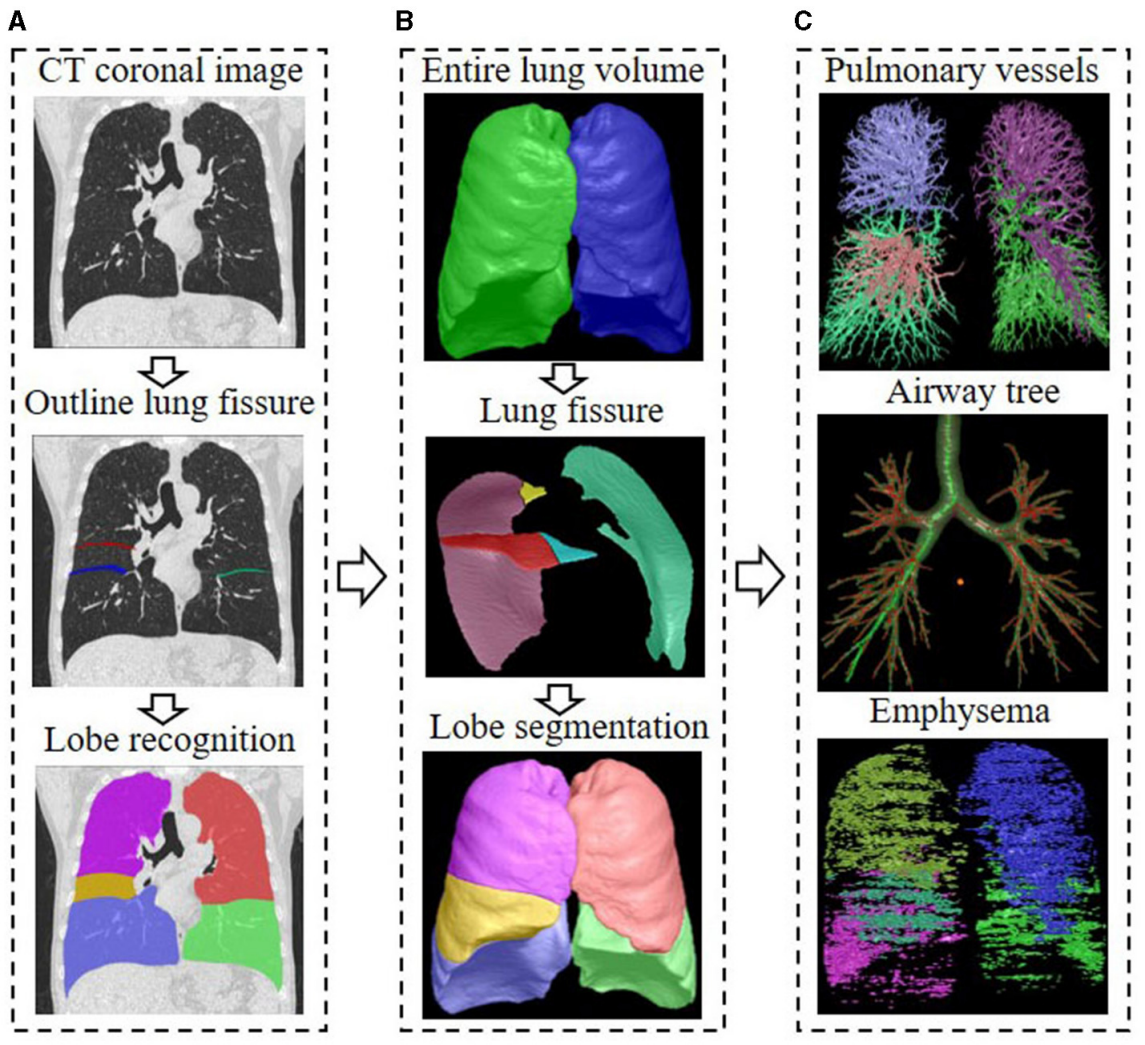
Flow chart of CT quantitative parameter measurement. **(A)** The delineation of lung fissures and the identification of lung lobes on CT images; **(B)** The total lung volume identified by the 3D adaptive border marching algorithm, the lung fissure segmented by the computational geometry approach, and the five lung lobes segmented by implicit surface functions; **(C)** The principal curvatures and the principal directions were used to distinguish pulmonary vessels from lung tissue, and the vascular tree was automatically extracted and segmented to calculate IPVV in the whole lung and each individual lobe; The differential geometric approach to segment the airway tree, and the average values of the measurements for the 3–6th bronchial generation were automatically calculated; The extent of emphysema under the threshold of −950 HU was automatically computed, the area shown in color.

We analyzed the CT scans using the FACT-Digital Lung Workstation (Dexin, Xi'an, China), which have both US FDA 510 K and CFDA cleared. This software system enables automated segmentation of a variety of lung structures, including right/left lungs, lung vessels, airway trees, inner/outer airway walls. On the basis of the segmentations, an automatically 3D approach was used to reconstruct the pulmonary vasculature and calculate the entire volume of the intrapulmonary vascular volume (IPVV) in the whole lung or each individual lobe. In inspiratory and expiratory CT, the measures of IPVV all includes the vascular wall and lumen of both arteries and veins, which is specified in milliliter (ml). We also measured the airway wall thickness (WT), and the percentage of the airway wall area (WA%) of the 3–6th generations and the extent of emphysema in each individual lobe of both inspiratory and expiratory CT examinations. The extent of emphysema, which is defined as the percentage of lung area with CT attenuation values < -950 HU at inspiration (LAA%_−950_), was also automatically computed at a threshold of −950 Hounsfield Unit (HU). The difference in the values between inspiratory and expiratory scans was defined as difference value, the ratio of inspiratory scans to expiratory was defined as relative value. Detailed descriptions of these computerized schemes have been reported elsewhere (22–24), and the segmentation results were shown in Figure 1.

### Statistical Analysis

We assessed the correlations of IPVV with PFT measures, WT_3−6_, and LAA%_−950_ using Pearson or Spearman's rank correlation analysis and multiple linear regression analysis with step-wise selection method for inspiratory and expiratory CT. Continuous data were tested for normality using the Shapiro-Wilk or Kolmogorov-Smirnov test according to the number of subjects. Data meeting the normal distribution were expressed as mean ± SD. Non-normally distributed data were expressed as median (interquartile range). The comparison of IPVV between mild COPD (GOLD stage I and II) and severe COPD (GOLD stage III and IV) was analyzed using the Student's *t*-test or Mann-Whitney *U*-test. Statistical analysis was performed using SPSS 20.0. A *p*-value < 0.05 was considered statistically significant.

## Results

**Figure 2:**
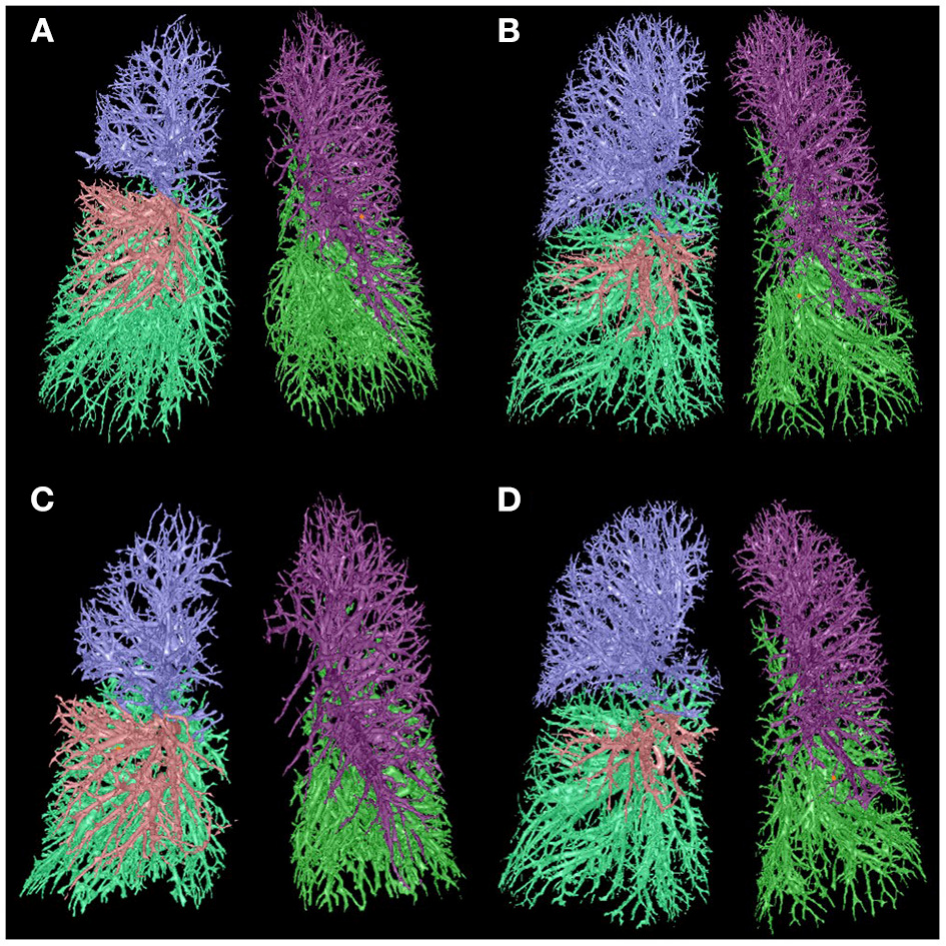
Comparison of IPVV between mild and severe COPD in inspiratory and expiratory CT. **(A,C)** A male with mild COPD (GOLD II, 68 years): a, the IPVV of the whole lung in inspiratory CT is 209.95 ml; b, the IPVV of the whole lung in expiratory CT is 169.10 ml. **(B,D)** A male with severe COPD (GOLD III, 64 years): a, the IPVV of the whole lung in inspiratory CT is 208.31 ml; b, the IPVV of the whole lung in expiratory CT is 201.18 ml.

**Figure 3:**
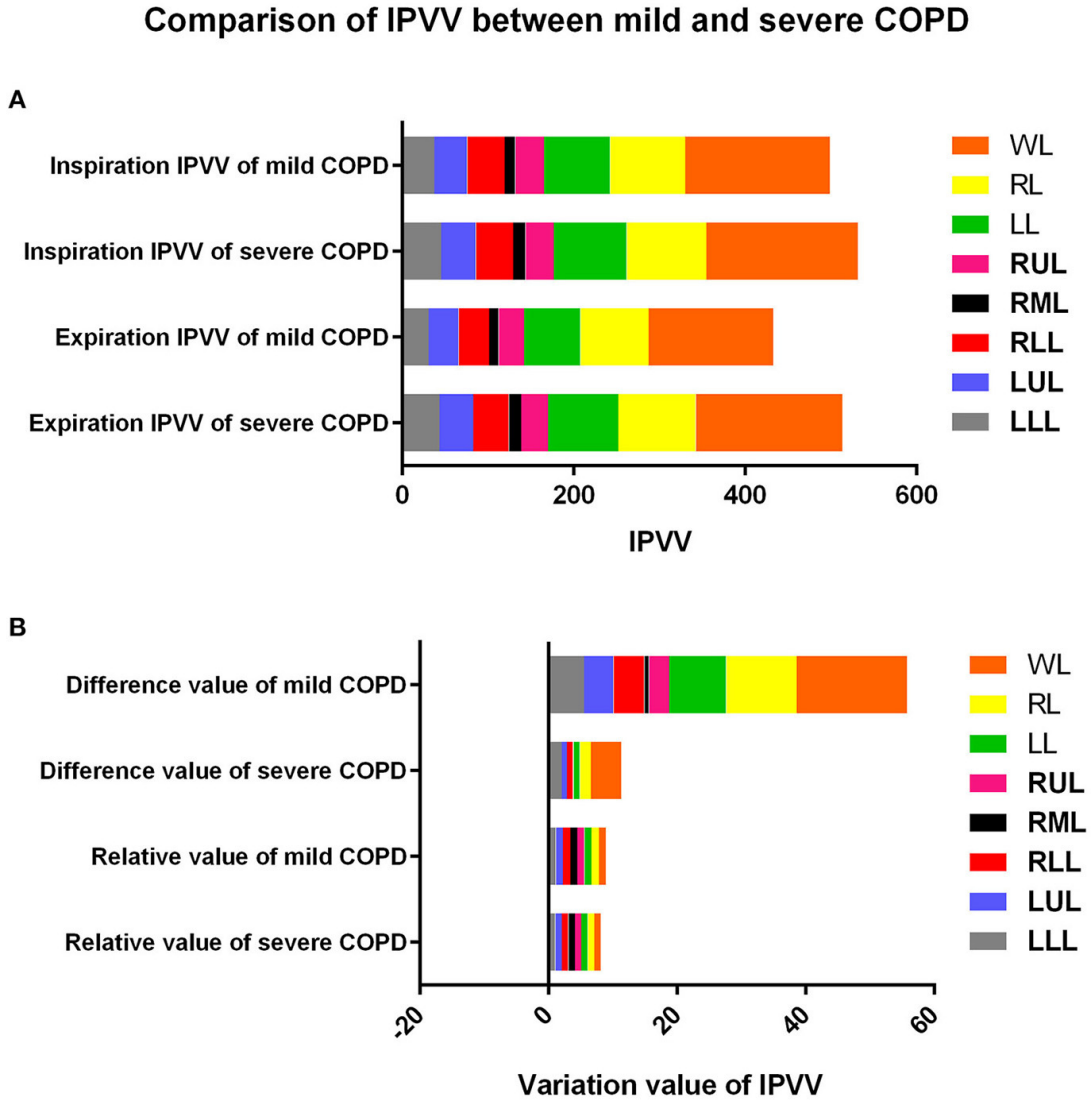
Comparison of IPVV between mild and severe COPD in inspiratory, expiratory CT, difference value and relative value. **(A)** Comparison in inspiratory and expiratory CT; **(B)** Comparison in difference value and relative value.

The comparison of IPVV between mild and severe COPD in inspiratory and expiratory CT were summarized in Table 2 and Figures 2, 3. In the subgroup with only expiratory CT examinations, there were significant differences of IPVV between mild and severe COPD groups, except for the right upper lobe (RUL, *p* = 0.286) and left upper lobe (LUL, *p* = 0.106). In contrast, in the subgroup with only inspiratory CT examinations, only the IPVV value in the left lower lobe (LLL, *p* = 0.006) showed a difference regardless COPD severity. The IPVV values of the lower lobes were consistently higher than those of the upper lobes in both inspiratory and expiratory CT scans. For the difference values and relative values, the changes of IPVV in the severe COPD groups were significantly less than the mild.

**Table 2:** Comparisons of IPVV between mild and severe COPD.

		**Mild COPD (*n* = 43)**	**Severe COPD (*n* = 46)**	***t*/*z*-value**	***P*-value**
**Inspiration IPVV**					
	WL	168.94 ± 44.12	176.78 ± 48.61	−0.796	0.428
	RL	87.66 (35.42)	92.83 ± 25.05	−0.452	0.652
	LL	77.12 ± 22.36	85.15 ± 24.04	−1.628	0.107
	RUL	33.68 (15.16)	33.07 (15.26)	−0.164	0.87
	RML	12.36 (5.69)	14.13 ± 5.42	−1.355	0.176
	RLL	43.41 ± 12.46*	43.69 ± 13.11*	−0.105	0.917
	LUL	38.37 (14.95)	40.29 ± 12.98	−0.435	0.663
	LLL	37.58 ± 13.24	45.65 ± 13.84	−2.809	0.006
**Expiration IPVV**					
	WL	145.37 ± 49.68	171.18 ± 45.11	−2.568	0.012
	RL	79.46 ± 25.73	90.46 ± 22.90	−2.133	0.036
	LL	65.91 ± 26.06	81.93 ± 22.98	−3.081	0.003
	RUL	29.57 (16.28)	31.50 (11.32)	−1.067	0.286
	RML	10.87 (5.00)	14.32 ± 5.54	−2.451	0.014
	RLL	35.69 ± 15.26	41.58 ± 12.56*	−1.993	0.049
	LUL	34.97 ± 13.99	39.53 ± 12.33	−1.635	0.106
	LLL	30.94 ± 16.01	43.34 ± 14.83	−3.793	<0.001
**Difference Value**					
	WL	17.19 (32.77)	4.72 (16.95)	−3.966	<0.001
	RL	11.00 (16.12)	1.71 (10.60)	−3.834	<0.001
	LL	8.82 (13.97)	1.00 (8.16)	−3.53	<0.001
	RUL	3.15 (4.82)	0.10 (3.23)	−3.875	<0.001
	RML	0.72 (1.76)	−0.19 ± 1.24	−3.654	<0.001
	RLL	4.80 (12.39)*	0.93 (5.09)	−3.296	0.001
	LUL	4.58 ± 5.60	0.78 (3.82)	−3.851	<0.001
	LLL	5.54 (7.52)	2.07 (6.17)	−3.206	0.001
**Relative Value**					
	WL	1.10 (0.24)	1.03 (0.10)	−4.335	<0.001
	RL	1.14 (0.25)	1.02 (0.12)	−4.171	<0.001
	LL	1.11 (0.30)	1.02 (0.09)	−3.966	<0.001
	RUL	1.10 (0.21)	1.00 (0.10)	−3.982	<0.001
	RML	1.09 (0.13)	1.01 (0.11)	−3.752	<0.001
	RLL	1.14 (0.36)	1.02 (0.12)	−3.465	0.001
	LUL	1.11 (0.20)	1.02 (0.10)	−3.998	<0.001
	LLL	1.14 (0.55)	1.04 (0.13)	−3.563	<0.001

IPVV, intrapulmonary vascular volume; COPD, chronic obstructive pulmonary disease; WL, the whole lung; RL, the right lung; LL, the left lung; RUL, the right upper lobe; RML, the right middle lobe; RLL, the right lower lobe; LUL, the left upper lobe; LLL, the left lower lobe. *Difference of IPVV between RUL and RLL, P < 0.05.

**Figure 4:**
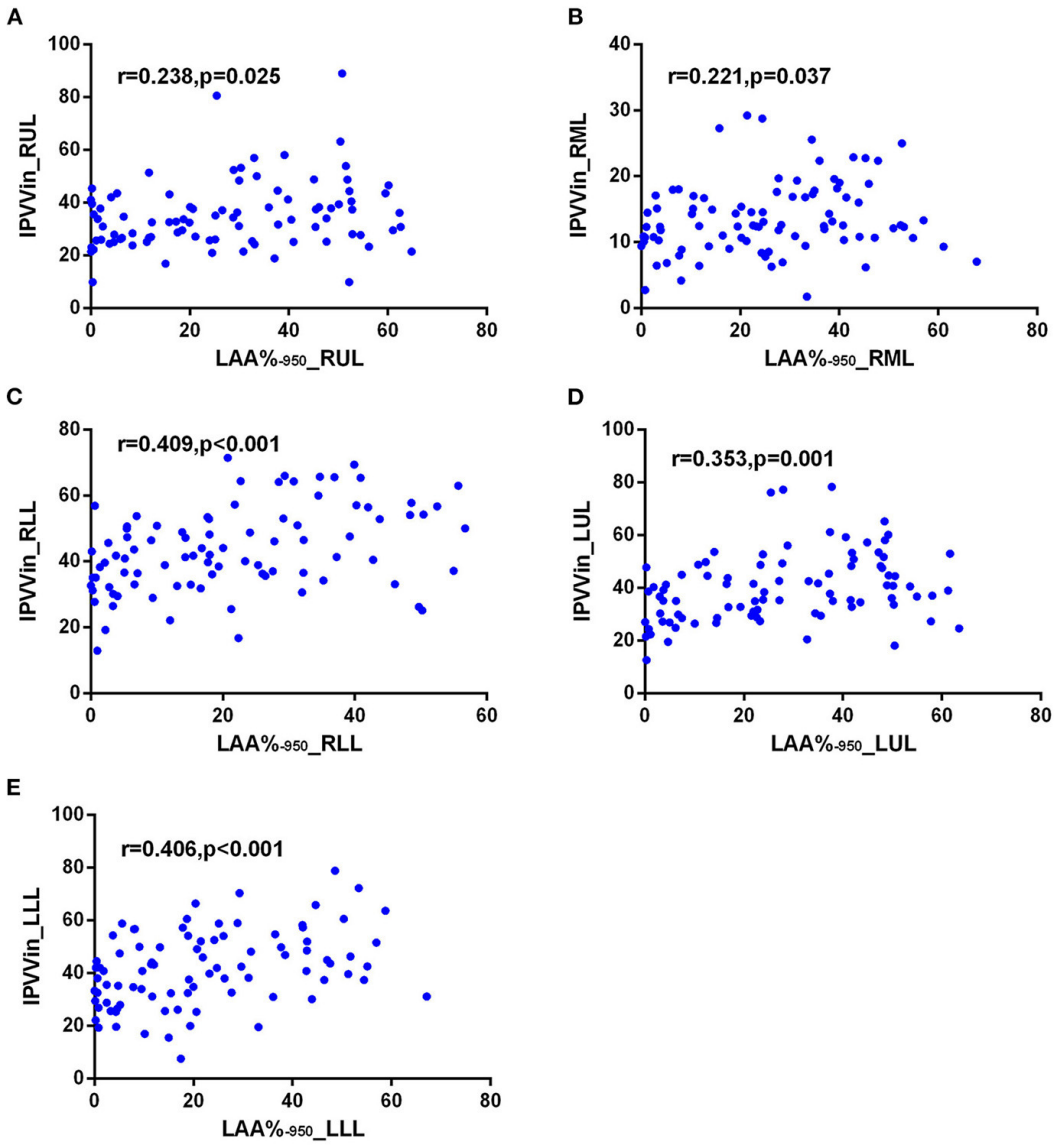
Correlations of IPVV in individual lobes with LAA%_−950_ in the inspiratory CT scan. **(A)** RUL; **(B)** RML; **(C)** RLL; **(D)** LUL; **(E)** LLL.

**Figure 5:**
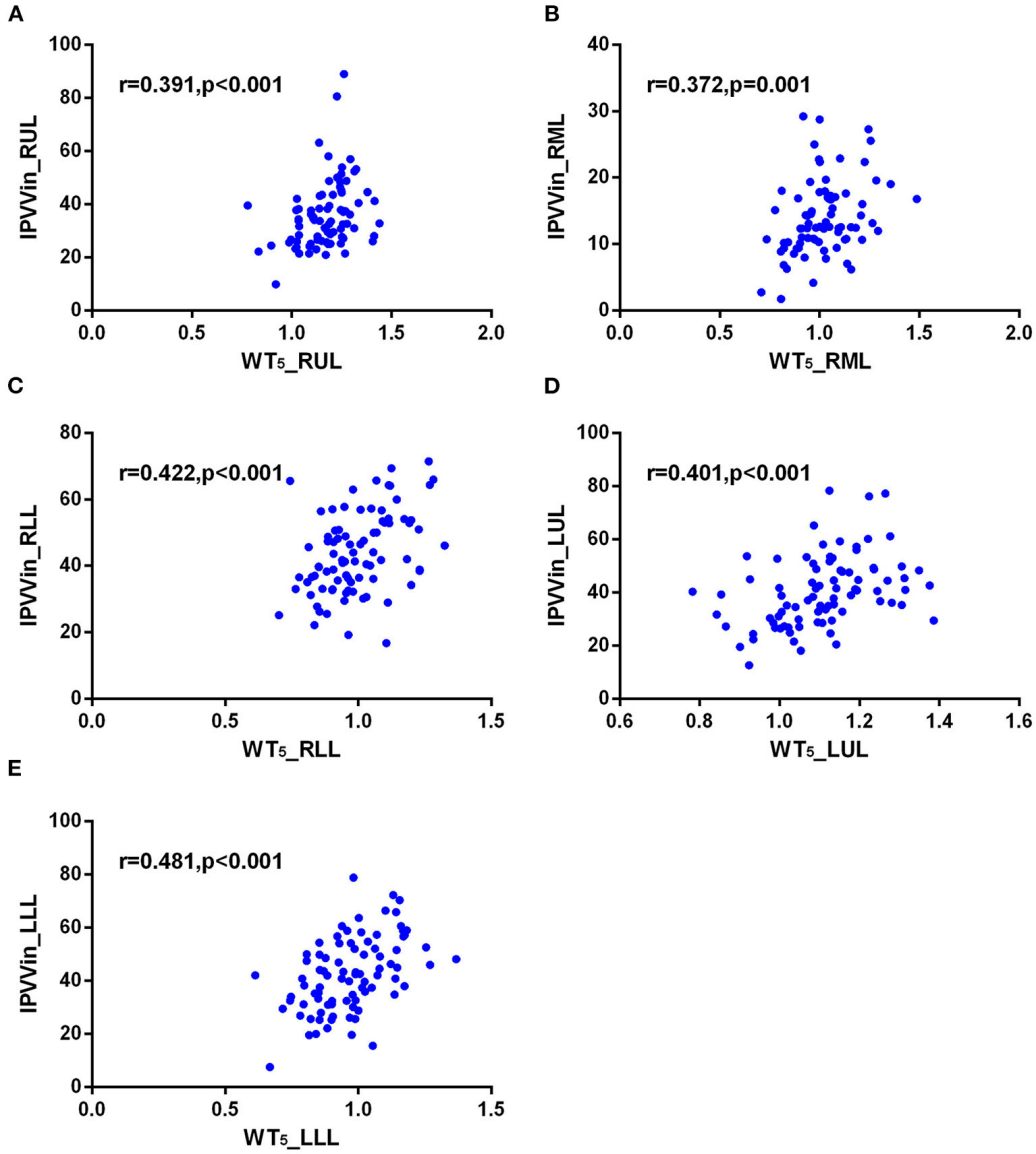
Correlations of IPVV in individual lobes with WT_5_ in the inspiratory CT scan. **(A)** RUL; **(B)** RML; **(C)** RLL; **(D)** LUL; **(E)** LLL.

The correlations between IPVV and PFT measures were presented in Table 3. For the inspiratory CT scan, there were mild negative correlations between IPVV and FEV_1_/FVC in each individual lobes (*r* = −0.215 to −0.292, all *p* < 0.05), between IPVV and FEV_1_% in right middle lobe (RML, *r* = −0.246, *p* = 0.020) and LLL(*r* = −0.230, *p* = 0.030). LAA%_−950_ (*r* = 0.221 to 0.409, all *p* < 0.05) and WT_3−6*th*_ (*r* = 0.233 to 0.557, all *p* < 0.05) were significantly associated with IPVV in all lobes (see Figures 4, 5 and Table 4). In particular, the strongest correlation was consistently observed for right lower lobe (RLL) and LLL. IPVV had no association WA%, except for WA%_4−5th_ in RML (*r* = −0.272, −0.236, respectively, *p* < 0.05) and WA%_6th_ in LUL(*r* = −0.219, *p* = 0.045).

**Table 3:** Correlation between IPVV and PFT.

**Pulmonary vascular measurement**	**Spirometry**	
	**FEV_**1**_/FVC**	**FEV_**1**_%**
**Inspiration**		
IPVV_RUL_	−0.289 (0.006)	−0.046 (0.666)
IPVV_RML_	−0.280 (0.008)	−0.246 (0.020)
IPVV_RLL_	−0.215 (0.043)	−0.062 (0.562)
IPVV_LUL_	−0.283 (0.007)	−0.094 (0.383)
IPVV_LLL_	−0.292 (0.005)	−0.230 (0.030)
**Expiration**		
IPVV_RUL_	−0.318 (0.002)	−0.168 (0.117)
IPVV_RML_	−0.346 (0.001)	−0.360 (0.001)
IPVV_RLL_	−0.238 (0.024)	−0.202 (0.057)
IPVV_LUL_	−0.326 (0.002)	−0.243 (0.022)
IPVV_LLL_	−0.292 (0.005)	−0.297 (0.005)
**Difference Value**		
IPVV_RUL_	0.157 (0.143)	0.393 (<0.001)
IPVV_RML_	0.137 (0.202)	0.353 (0.001)
IPVV_RLL_	0.176 (0.099)	0.338 (0.001)
IPVV_LUL_	0.162 (0.130)	0.406 (<0.001)
IPVV_LLL_	0.173 (0.106)	0.311 (0.003)
**Relative Value**		
IPVV_RUL_	0.200 (0.060)	0.412 (<0.001)
IPVV_RML_	0.211 (0.047)	0.391 (<0.001)
IPVV_RLL_	0.197 (0.064)	0.367 (<0.001)
IPVV_LUL_	0.226 (0.033)	0.444 (<0.001)
IPVV_LLL_	0.228 (0.032)	0.359 (0.001)

*FEV_1_/FVC, ratio of forced expiratory volume in 1 s to forced vital capacity; FEV_1_, percentage predicted forced expiratory volume in 1 s; IPVV, the intrapulmonary vascular volume; All P-values were presented in parentheses*.

**Table 4:** Correlations between IPVV and other parameters.

**Pulmonary vascular measurement**	**Emphysema index**	**Airway measurements**							
	**LAA%–_**950**_**	**WA%_**3**_**	**WA%_**4**_**	**WA%_**5**_**	**WA%_**6**_**	**WT_**3**_**	**WT_**4**_**	**WT_**5**_**	**WT_**6**_**
**Inspiration**									
IPVV_RUL_	0.238 (0.025)	−0.099 (0.362)	−0.124 (0.256)	−0.062 (0.575)	−0.157 (0.176)	0.233 (0.030)	0.257 (0.017)	0.391 (<0.001)	0.415 (<0.001)
IPVV_RML_	0.221 (0.037)	−0.142 (0.192)	−0.272 (0.013)	−0.236 (0.037)	−0.101 (0.437)	0.280 (0.009)	0.258 (0.019)	0.372 (0.001)	0.257 (0.043)
IPVV_RLL_	0.409 (<0.001)	−0.109 (0.311)	0.045 (0.684)	0.100 (0.364)	0.219 (0.045)	0.448 (<0.001)	0.460 (<0.001)	0.422 (<0.001)	0.557 (<0.001)
IPVV_LUL_	0.353 (0.001)	−0.027 (0.802)	−0.046 (0.678)	0.031 (0.776)	−0.021 (0.852)	0.383 (<0.001)	0.351 (0.001)	0.401 (<0.001)	0.425 (<0.001)
IPVV_LLL_	0.406 (<0.001)	−0.036 (0.745)	0.079 (0.470)	0.181 (0.096)	0.087 (0.434)	0.440 (<0.001)	0.487 (<0.001)	0.481 (<0.001)	0.432 (<0.001)
**Expiration**									
IPVV_RUL_	0.265 (0.012)	0.137 (0.218)	−0.030 (0.793)	−0.144 (0.248)	0.073 (0.589)	0.370 (0.001)	0.394 (<0.001)	0.422 (<0.001)	0.418 (0.001)
IPVV_RML_	0.241 (0.023)	−0.160 (0.157)	−0.186 (0.124)	−0.167 (0.213)	−0.093 (0.631)	0.308 (0.005)	0.260 (0.030)	0.242 (0.070)	0.256 (0.181)
IPVV_RLL_	0.362 (<0.001)	−0.027 (0.807)	−0.091 (0.425)	0.153 (0.184)	0.158 (0.182)	0.529 (<0.001)	0.383 (<0.001)	0.527 (<0.001)	0.504 (<0.001)
IPVV_LUL_	0.361 (0.001)	0.040 (0.717)	0.088 (0.437)	−0.051 (0.665)	−0.048 (0.718)	0.417 (<0.001)	0.455 (<0.001)	0.446 (<0.001)	0.566 (<0.001)
IPVV_LLL_	0.355 (0.001)	−0.266 (0.016)	0.139 (0.238)	−0.178 (0.140)	0.199 (0.146)	0.474 (<0.001)	0.441 (<0.001)	0.481 (<0.001)	0.535 (<0.001)

*IPVV, the intrapulmonary vascular volume; LAA%_−950_, the percentage of lung area with CT attenuation values < -950HU; WT_3−6_, airway wall thickness of the 3–6th generations; WA%_3−6_, the percentage of the airway wall area of the 3–6th generations. All P-values were presented in parentheses*.

**Figure 6:**
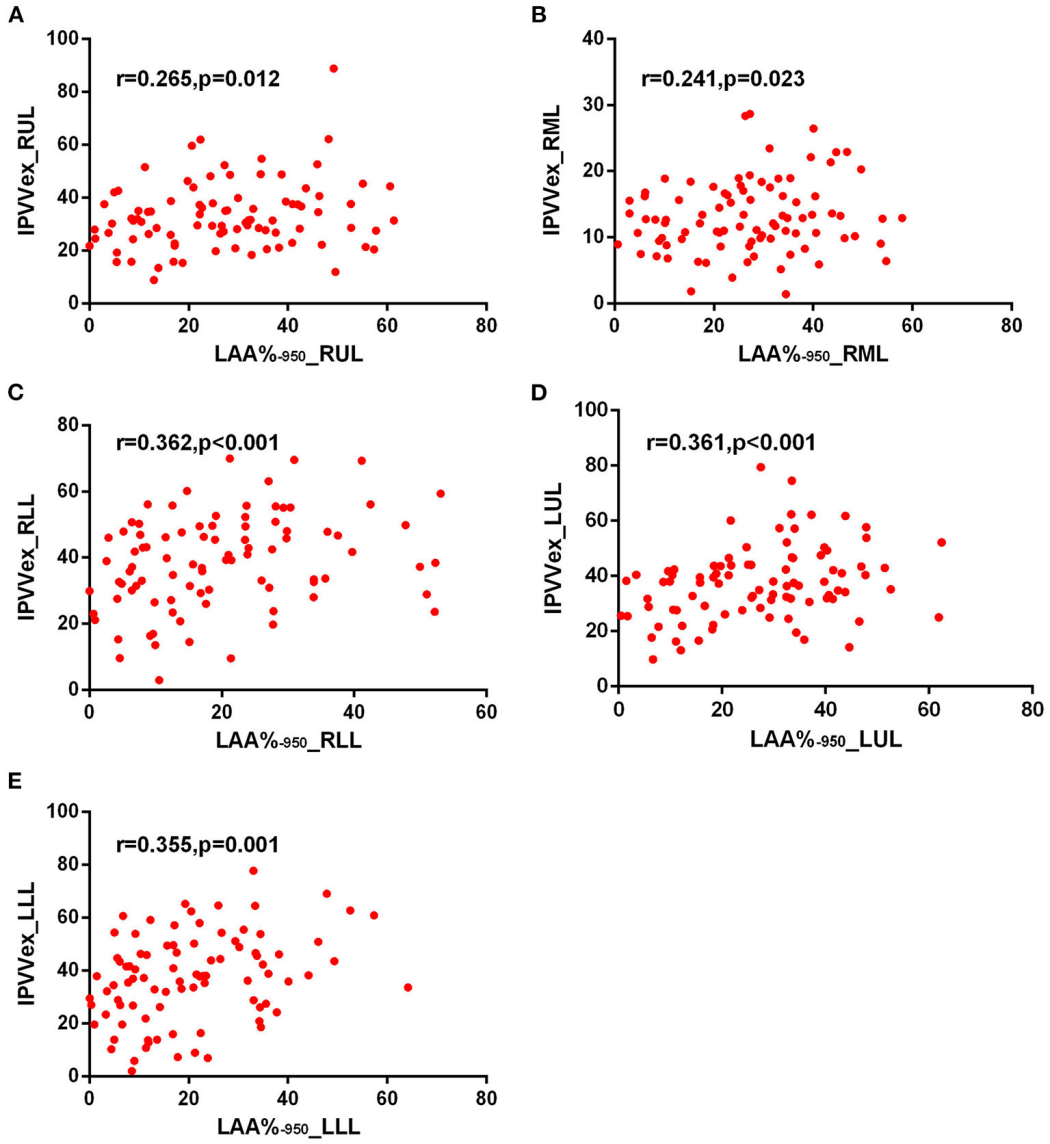
Correlations of IPVV in individual lobes with LAA%_−950_ in the expiratory CT scan. **(A)** RUL; **(B)** RML; **(C)** RLL; **(D)** LUL; **(E)** LLL.

**Figure 7:**
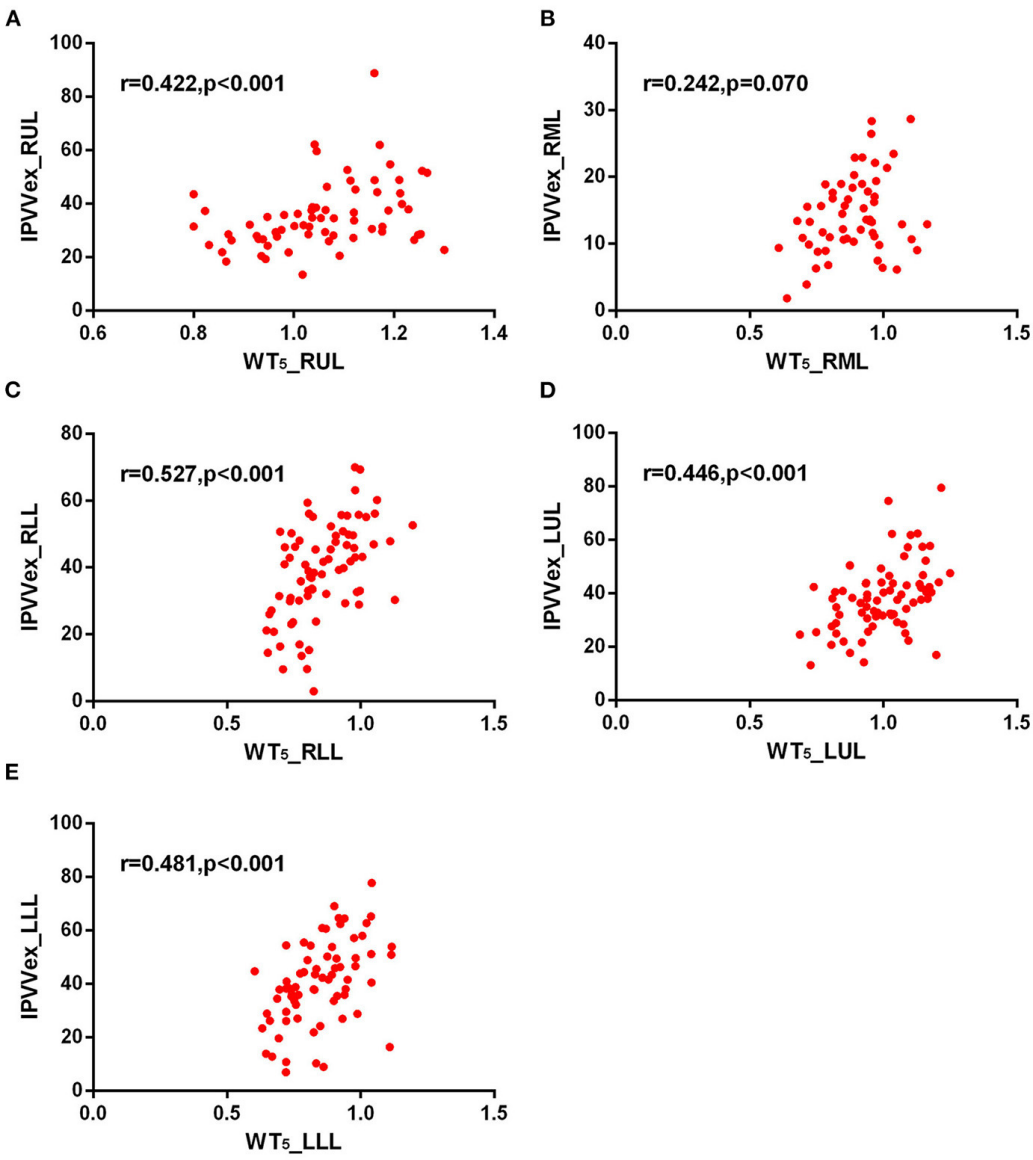
Correlations of IPVV in individual lobes with WT_5_ in the expiratory CT scan. **(A)** RUL; **(B)** RML; **(C)** RLL; **(D)** LUL; **(E)** LLL.

For expiratory CT scans, FEV_1_/FVC (*r* = −0.238 to −0.346, *p* < 0.05) and FEV_1_% (*r* = −0.243 to −0.360, all *p* < 0.05) had a significant, mild-to-moderate negative correlation with IPVV, except for FEV_1_% in RUL and RLL. LAA%_−950_ and WT_3−6*th*_ (except for WT_5−6th_ in RML) positively correlated with IPVV (see Figures 6, 7 and Table 4). Similar to the inspiratory CT, IPVV had no association with WA%, except for WA%_3th_ (*r* = −0.266, *p* = 0.016) in LLL. The correlation coefficients of the expiratory CT were slightly higher than that of the inspiratory CT.

For the respiratory variation, FEV_1_% showed significant, moderate positive correlations with the difference value and relative value (*r* = 0.350–0.463, all *p* < 0.05), and FEV_1_/FVC showed mild positive correlations with the relative value (except for RUL and RLL, *p* > 0.05). There was no correlation between FEV_1_/FVC and the difference value.

Table 5 shows the results of multiple linear regression analysis, where IPVV was the dependent variable, age, BMI and other CT parameters were the independent variables. In inspiratory CT, the *R*^2^ values of each pulmonary lobe regression equation were within the range of 0.075–0.426, while the *R*^2^ was 0.165–0.559 in the expiratory, except for RML. The *R*^2^ value of each lobe in the expiratory was higher than the inspiratory. The largest *R*^2^ was observed at LLL in two respiratory phases, while the least is RML. The multiple regression analysis for IPVV revealed that WT was a significant independent predictor of IPVV at the inspiratory and expiratory CT, particularly in difference value and relative value.

**Table 5:** Multiple linear regression analysis of IPVV in inspiratory CT, expiratory CT, difference value and relative value.

**Inspiratory IPVV**		**β**	**CI**	***P*-value**
RUL (*R*^2^ = 0.160)	BMI	−0.88	(−1.75, −0.02)	0.045
	WT_6_	39.86	(14.95, 64.77)	0.002
RML (*R*^2^ = 0.075)	WT_5_	10.05	(0.40, 19.69)	0.042
RLL (*R*^2^ = 0.373)	LAA%–_950_	0.21	(0.06, 0.35)	0.006
	WT_6_	46.45	(27.91, 65.00)	<0.001
LUL (*R*^2^ = 0.189)	WT_5_	46.80	(24.17, 69.44)	<0.001
LLL (*R*^2^ = 0.426)	Age	−0.38	(−0.66, −0.11)	0.007
	LAA%–_950_	0.18	(0.04, 0.33)	0.016
	WA%_4_	−46.00	(−81.93, −10.07)	0.013
	WT_4_	50.17	(30.98, 69.37)	<0.001
**Expiratory IPVV**		***β***	**CI**	***P*** **-value**
RUL (*R*^2^ = 0.165)	WT_6_	41.22	(15.11, 67.33)	0.003
RLL (*R*^2^ = 0.439)	Age	−0.32	(−0.63, −0.01)	0.045
	LAA%–_950_	0.29	(0.08, 0.50)	0.007
	WA%_4_	−61.44	(−108.07, −14.81)	0.011
	WT_5_	67.73	(43.21, 92.24)	<0.001
LUL (*R*^2^ = 0.330)	WT_5_	61.69	(37.69, 85.68)	<0.001
LLL (*R*^2^ = 0.559)	Age	−0.50	(−0.83, −0.16)	0.005
	WA%_3_	−95.39	(−144.02, −46.75)	0.001
	WT_6_	67.83	(41.88, 93.79)	<0.001
	LAA%–_950_	0.24	(0.04, 0.45)	0.021
**Difference Value**		***β***	**CI**	***P*** **-value**
RUL (*R*^2^ = 0.196)	WT_6_	18.42	(7.60, 29.24)	0.001
RML (*R*^2^ = 0.174)	WT_3_	4.97	(0.30, 9.65)	0.038
RLL (*R*^2^ = 0.247)	WT_5_	27.92	(15.73, 40.10)	<0.001
LUL (*R*^2^ = 0.191)	WT_4_	14.05	(5.93, 22.17)	0.001
LLL (*R*^2^ = 0.283)	Age	0.22	(0.04, 0.41)	0.017
	WT_4_	22.50	(10.71, 34.29)	<0.001
**Relative Value**		***β***	**CI**	***P*** **-value**
RUL (*R*^2^ = 0.359)	WT_6_	0.39	(0.03, 0.76)	0.035
	LAA%–_950_	−0.11	(−0.19, −0.04)	0.004
	WT_4_	0.40	(0.03, 0.76)	0.035
RML (*R*^2^ = 0.385)	WT_3_	0.79	(0.32, 1.26)	0.002
	WA%_3_	−0.93	(−1.70, −0.17)	0.019
RLL (*R*^2^ = 0.119)	WT_5_	1.54	(0.49, 2.59)	0.005
LUL (*R*^2^ = 0.253)	WT_3_	0.49	(0.25, 0.72)	<0.001
LLL (*R*^2^ = 0.311)	WT_5_	1.47	(0.79, 2.15)	<0.001
	WA%_5_	−1.11	(−1.78, −0.45)	0.002

*BMI, body mass index; IPVV, the intrapulmonary vascular volume; LAA%_−950_, the percentage of lung area with CT attenuation values < -950 HU; WT_3−6_, airway wall thickness of the 4–6th generations; WA%_3_-_4_, the percentage of the airway wall area of the 3–4th generations*.

## Discussion

In this study, we quantified IPVVs depicted on both inspiratory and expiratory CT scans and investigated their associations with pulmonary functions, airway remodeling, and disease severity in COPD patients. The analyses were performed at the levels of the entire lungs and individual lobes. Our experimental results showed that the IPVV could serve as a quantitative index for pulmonary vascular alternations in COPD patients. In particular, the IPVVs quantified using expiratory CT examinations does not only provide a more reliable and accurate assessment of pulmonary vascular alternations and COPD as well as their progression than the inspiratory CT examinations, but also allows the calculation of the difference and relative value to show the dynamic changes of IPVV during respiration. Additionally, the multiple linear regression analyses showed that bronchial wall thickness had significant correlation with IPVV and suggested that WT might be an independent predictor of pulmonary vascular alteration in COPD.

Our findings are in consistent with Estepar et al.'s investigation (25), where there was no association between COPD severity and total blood vessel volume depicted on inspiratory CT scans. In contrast, in the expiratory CT examinations, the IPVV in severe COPD subgroup were significantly higher than those in the mild COPD subgroup at the levels of in the entire lung and individual lobes except for RUL and LUL. The difference value and relative value between inspiratory and expiratory CT revealed that the alteration of severe COPD was less than the mild. The results demonstrated that pulmonary vascular alternations between breaths decreased with the increase of COPD severity.

Stronger correlations were found between IPVV and PFT in the expiratory CT compared with the inspiratory CT. This finding reinforces the viewpoint about the value of expiratory CT in COPD patients proposed by previous studies (17, 26). Matsuoka et al. (27) reported that the correlation coefficients between airway luminal area measured at expiratory CT and PFT were higher than those for inspiratory CT. Gawlitza et al. (17) demonstrated that quantitative CT parameters of emphysema such as mean lung density and low attenuation volume in expiratory phase show stronger correlation with lung function testing than the inspiratory. Nevertheless, there are few studies on the expiratory CT involving pulmonary blood vessels. Our study not only verified higher correlations in expiratory CT but also found significant correlations between FEV_1_% and the difference, relative values. Compared with the difference value, the relative value may be the better indicator of changes in pulmonary vessels during respiration. From a pathophysiological standpoint, this may be explained by promotion of pulmonary vasoconstriction and remodeling by expiratory state in patients with airflow obstruction (6).

We in particular investigated the association between pulmonary vascular disease and airway disease. Very limited investigations (28) have been conducted in this regard. We found that the airway wall thickness correlated positively with the IPVV on both inspiratory and expiratory CT. Our finding indicated that vascular alteration in COPD was influenced by both emphysema and airway remodeling, and the multiple linear regression analyses of inspiratory, expiratory CT, the difference and relative value showed that the main parameter able to explain pulmonary vascular alteration in patients with COPD was WT, which was similar in part to the result reported by Coste et al. (29). Furthermore, the higher correlations in the expiratory CT demonstrated that the expiratory CT had potential value in quantitative pulmonary vascular disease and evaluating the severity and progress of COPD, compared with the inspiratory CT.

When evaluating the distribution of pulmonary vascular alteration in different lobes, Wrobel et al. (30) quantified the percentage wall thickness to vessel diameter and showed that there was increased pulmonary arterial remodeling in the upper lobes compared with the lower lobes in subjects with COPD. Our results were in consistent with Estepar et al.'s (25) but contradict with Wrobel et al.'s (30) in that the IPVV of the lower lobes was higher than that of the upper lobes. However, this study failed to observe significant difference in IPVV between LUL and LLL. This may be due to the influence of cardiac motion in the left lung, resulting in some errors in IPVV measurement (31) and the limitation of the small datasets. Additional efforts are needed to verify this.

We are aware that the primary limitations with this study is the relatively small dataset for the analyses. There is significant imbalance with the study population in many aspects, such as gender, disease severity, and lung functions. All these along with other potential confounding factors (e.g., image quality and acquisition protocols) could unavoidably lead to some biases in both conclusion and analyses, and this may also be the reason why the correlation coefficient of this study is small. Nevertheless, the findings in this study suggest the unique potential of expiratory CT scans in analyzing pulmonary vascular alternations and the potential association of pulmonary vascular alternations with COPD and other airway diseases.

In conclusion, the quantitative parameter IPVV demonstrated significant associations with PFT, emphysema and airway disease in patients with COPD, the expiratory CT and the relative values showed potential values in quantifying pulmonary vascular alterations and evaluating the severity of COPD. Additionally, the airway wall thickness may be the independent predictor of pulmonary vascular alteration in COPD. Further work is required to clarify and validate the exact value of expiratory CT in quantitative pulmonary vessels in COPD with advanced quantitative technique.

## Data Availability Statement

The original contributions presented in the study are included in the article/supplementary material, further inquiries can be directed to the corresponding author/s.

## Ethics Statement

The studies involving human participants were reviewed and approved by the Chinese Clinical Research Registry (Grant No.: ChiCTR-OCH-14004904) and written informed consent was obtained from all subjects. The patients/participants provided their written informed consent to participate in this study.

## Author Contributions

XC, CJ, and YG conceived of the idea. XC conducted statistical analyses. XG, NY, XW, and XH collected the data. XC and XG wrote the manuscript with inputs from all authors. All authors contributed to the article and approved the submitted version.

## Funding

This work was supported by the National Institutes of Health from National Health and Family Planning Commission of China (No. 201402013).

## Conflict of Interest

The authors declare that the research was conducted in the absence of any commercial or financial relationships that could be construed as a potential conflict of interest.

## Publisher's Note

All claims expressed in this article are solely those of the authors and do not necessarily represent those of their affiliated organizations, or those of the publisher, the editors and the reviewers. Any product that may be evaluated in this article, or claim that may be made by its manufacturer, is not guaranteed or endorsed by the publisher.
